# Li-ion battery material under high pressure: amorphization and enhanced conductivity of Li_4_Ti_5_O_12_

**DOI:** 10.1093/nsr/nwy122

**Published:** 2018-10-29

**Authors:** Yanwei Huang, Yu He, Howard Sheng, Xia Lu, Haini Dong, Sudeshna Samanta, Hongliang Dong, Xifeng Li, Duck Young Kim, Ho-kwang Mao, Yuzi Liu, Heping Li, Hong Li, Lin Wang

**Affiliations:** 1 Center for High Pressure Science and Technology Advanced Research, Shanghai 201203, China; 2 Key Laboratory of High-temperature and High-pressure Study of the Earth's Interior, Institute of Geochemistry, Chinese Academy of Sciences, Guiyang 550081, China; 3 Department of Physics and Astronomy, George Mason University, Fairfax VA 22030, USA; 4 College of Materials and Environmental Engineering, Hangzhou Dianzi University, Hangzhou 310018, China; 5 State Key Laboratory of Organic-Inorganic Composites, Beijing Advanced Innovation Center for Soft Matter Science and Engineering, College of Energy, Beijing University of Chemical Engineering, Beijing 100029, China; 6 School of Mechatronic Engineering and Automation, Shanghai University, Shanghai 200072, China; 7 Geophysical Laboratory, Carnegie Institution, Washington, DC 20015, USA; 8 Center for Nanoscale Materials, Argonne National Laboratory, Argonne, IL 60439, USA; 9 Beijing National Laboratory for Condensed Matter Physics, Institute of Physics, Chinese Academy of Sciences, Beijing 100190, China

**Keywords:** high pressure, pressure-induced amorphization, lithium-ion battery materials, ionic conductivity

## Abstract

Lithium titanium oxide (Li_4_Ti_5_O_12_, LTO), a ‘zero-strain’ anode material for lithium-ion batteries, exhibits excellent cycling performance. However, its poor conductivity highly limits its applications. Here, the structural stability and conductivity of LTO were studied using *in situ* high-pressure measurements and first-principles calculations. LTO underwent a pressure-induced amorphization (PIA) at 26.9 GPa. The impedance spectroscopy revealed that the conductivity of LTO improved significantly after amorphization and that the conductivity of decompressed amorphous LTO increased by an order of magnitude compared with its starting phase. Furthermore, our calculations demonstrated that the different compressibility of the LiO_6_ and TiO_6_ octahedra in the structure was crucial for the PIA. The amorphous phase promotes Li^+^ diffusion and enhances its ionic conductivity by providing defects for ion migration. Our results not only provide an insight into the pressure depended structural properties of a spinel-like material, but also facilitate exploration of the interplay between PIA and conductivity.

## INTRODUCTION

Lithium-ion batteries (LIBs) have been regarded as one of the most important components of portable electronics in our daily life, electric vehicles, stationary power storage and so on [1,2]. Energy density, safety, cost and the performance of LIBs are key factors, which are all controlled by the materials used [3,4]. Lithiated metal oxides and carbonaceous materials are commonly used as positive and negative electrode materials. However, carbon-based anodes suffer from the formation of a solid electrolyte interface, which often causes poor rate performance and safety issues [5]. As an alternative material, lithium titanium oxide (Li_4_Ti_5_O_12_, LTO) has been studied extensively as an ideal LIBs material, because its Li-ion insertion process operates at ∼1.5 V, which ensures great safety characteristics. Furthermore, it shows a negligible volume change during the Li-ion intercalation–deintercalation processes and is called as a ‘zero-strain’ anode material for LIBs, exhibiting excellent cycling performance and making it a promising anode for LIBs [6–8]. However, LTO shows poor electronic and ionic conductivity [9,10], which limits its application. Therefore, improvement of its conductivity is crucial. The migration ability and open level of the immigration channels of Li^+^ are key factors, but at times they compete. For example, breaking the LiO_6_ octahedron in LTO will probably enhance the migration of Li^+^, but it could close the open channel for the ion and void the improvement under certain conditions. *In situ* investigations of transport properties during the process of amorphization will be beneficial for optimizing the performance of such materials.

Pressure has long been recognized as a powerful thermodynamic variable, which can tune properties of materials widely [11,12]. The pressure-induced amorphization (PIA) process that occurs in solids is very important in many fields, including Earth and planetary sciences, physics, chemistry and materials science[13–16]. The formation mechanism of PIA depends significantly on the types and structures of materials. The PIA taking place in solid H_2_O was ascribed to a density-driven phase transition. The isomorphism phenomenon with H_2_O was found earlier in other tetrahedrally coordinated solids, such as Si and GeO_2_, and also in octahedrally coordinated TiO_2_ [17–19]. A high-density amorphous (HDA) phase during the PIA process could transform into low-density amorphous (LDA) structures during decompression. Recently, high-energy pair distribution function (PDF) measurements confirmed the amorphization associated with the breakdown of the long-range order of the YO_6_ octahedron in Y_2_O_3_ [20]. The interlinking of the YO_6_ octahedron along *a*-*c* is much easier to break down compared to the connection along *a*-*b* for the Y_2_O_3_ crystal. In contrast to the polyhedra in the Y_2_O_3_ crystal, the octahedrally coordinated spinel Li_4_Ti_5_O_12_ involves two different octahedra and two types of interlinking. We expected a new candidate for studying PIA in Li_4_Ti_5_O_12_ with two kinds of polyhedra—LiO_6_ and TiO_6_ octahedra—and were thus motivated to expand our research of the amorphization mechanism and related macroscopic properties. A thorough study of the crystal structure evolution and new phase formation can improve our understanding of the structural properties of this important anode material. Moreover, high-pressure-induced phase transitions have been observed in other LIB materials such as LiFePO_4_, LiMn_2_O_4_ and Li_2_MnSiO_4_ [21–24]. These high-pressure polymorphs may become novel materials for LIBs.

With the above-mentioned motivations, we investigated the structural stability and conductance of spinel LTO under high pressure up to 50 GPa. The pressure-induced amorphization pressure started at 26.9 GPa. Our calculations reveal that the different compressibility of the LiO_6_ and TiO_6_ octahedra in LTO is the key to the transition process. More importantly, ac impedance spectroscopy showed that the conductivity of LTO was greatly enhanced in its amorphous state, which offers us a new clue to overcoming the well-known issue of LTO’s poor conductivity.

## RESULTS AND DISCUSSION

**Figure 1.**
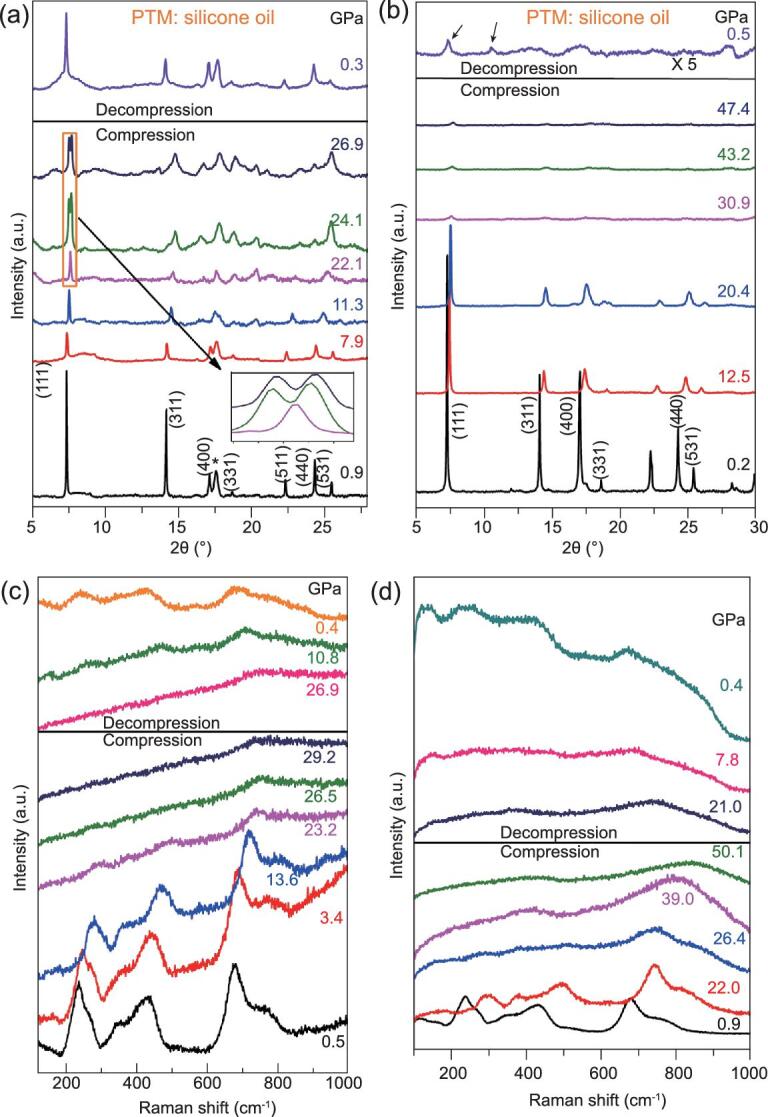
XRD patterns and Raman spectra of LTO spinel under compression and decompression. (a) Exerting pressure up to 26.9 GPa. The asterisk indicates the diffraction peak from the gasket. The inset shows magnified (111) peaks. (b) Exerting pressure up to 47.4 GPa. (c) Raman spectra in the range of 0.5∼29.2 and (d) 0.9∼50.1 GPa.

Figure 1a shows the evolution of the x-ray diffraction (XRD) patterns for the LTO spinel upon compression and decompression using silicone oil as a pressure-transmitting medium (PTM). The diffraction peaks identified in the starting structure were consistent with the diffraction pattern of cubic LTO. Figure 1a shows that the intensity of all the diffraction peaks became significantly weaker and the broadening of the peaks became more obvious between 0.9 and 26.9 GPa during compression. At 26.9 GPa, the (111) peak split into two peaks, which is magnified in the inset of Fig. 1a, indicating a transformation of the crystalline LTO structure. However, the diffraction pattern could not be indexed to any space group. Our further calculations using density function theory (see Supplementary Materials for details, available as Supplementary Data at *NSR* online) suggest the splitting is due to the lattice distortion of the spinel. Upon decompression, the LTO spinel recovered when the pressure was released to 0.3 GPa. This distorted phase was metastable and transformed back into the LTO spinel when the stress was released. The LTO spinel showed a plausibly reversible process when the maximum external pressure was 26.9 GPa. To ascertain the structural properties under higher pressure, we applied pressure on the starting spinel phase to a maximum value of 47.4 GPa. Figure 1b shows the pressure dependence of the XRD patterns for the LTO spinel. The sample maintained its initial phase well until the pressure increased to 20.4 GPa. Then, the diffraction peaks weakened and remarkable peak shifts to a large angle were observed because of the reduction in the lattice parameters. We observed peak splitting again at 25.3 GPa. All the diffraction peaks then started to disappear, indicating the loss of the long-range ordered structure, thus indicating that PIA had occurred. The phase remained amorphous at pressures up to 47.4 GPa. When the pressure was released to 0.5 GPa, the phase did not transform back into crystalline LTO, and only a few weak peaks were detected (marked with arrows), as shown in Fig. 1b. This phenomenon is similar to the PIA process found in nano-TiO_2_ [14–17]. The existence of several weak peaks might suggest the formation of nanopolycrystals dispersed in the LDA phase.

*In situ* Raman spectroscopy was employed to characterize the changes in the local structure of the LTO spinel under high pressure. Figure 1c shows the Raman spectra of the LTO spinel under compression from ambient pressure to 29.2 GPa. These Raman bands are features of the spinel structure (A_1_*_g_*+E*_g_*+3F_2_*_g_*) for LTO. The high-frequency Raman bands at 674 and 759 cm^−1^ can be assigned to the vibrations of the Ti–O bonds in the TiO_6_ octahedra; the middle frequency bands at 344 and 425 cm^−1^ are from the stretching vibrations of the Li–O bonds in the LiO_6_ polyhedra; and the low-frequency peaks are due to the bending vibrations of O-Ti-O and O-Li-O, respectively. Supplementary Fig. 3a–f, available as Supplementary Data at *NSR* online, show the Raman spectra at different pressures and the peak positions and intensities as a function of pressure. It is clear that all the Raman bands show linear blue shifts up to 21.8 GPa, suggesting the two octahedra are compressed monotonically and no crystal structure change occurs, which agrees with the XRD results. The spinel structure was preserved from 0.5 to 21.8 GPa. The intensity of the peaks became weak and sharply decreased as pressure reached ∼23.2 GPa, suggesting the structure coherence reduced considerably due to lattice distortion, in agreement with the XRD observation. When the pressure was increased further to 26.5 GPa, the peaks almost disappeared, except for the weak peak at 674 cm^−1^, indicating an obvious phase transition to an amorphous state. Upon decompression, the typical Raman features of crystalline LTO started to emerge at pressures below 10.8 GPa. The LTO-spinel phase could be fully restored when the pressure was released to 0.4 GPa. These results were fairly consistent with the results obtained from XRD. To investigate the PIA effect of LTO further, another *in situ* Raman spectroscopy measurement was performed from ambient pressure to a higher uppermost pressure of 50.1 GPa. The disappearance of all the peaks was also observed in this measurement at pressures above 26.4 GPa, confirming the amorphization of LTO (Fig. 1d). The weak broad peak near 690–788 cm^−1^ blue-shifted by increasing pressure from 26.4 to 50.1 GPa. No new phase transition was observed in this pressure range. When the pressure was released to ambient pressure, new broad features began to emerge, confirming a dominant amorphous phase. However, very weak Raman signals of LTO still existed in the spectrum, indicating the formation of leftover nanopolycrystals dispersed in the LDA phase, which is also consistent with the XRD results.

**Figure 2.**
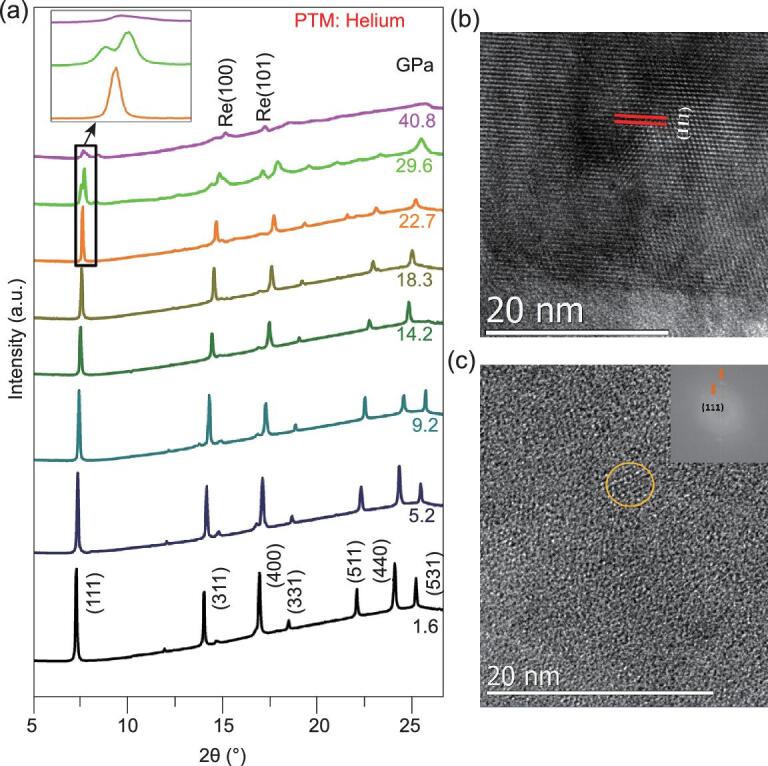
(a) XRD patterns of the LTO spinel under compression using helium as a PTM. The weak diffraction peaks from rhenium are marked. HRTEM images of the starting (b) and recovered samples (c).

To examine the effects of pressure inhomogeneity on the PIA, hydrostatic compression using helium as a PTM and non-hydrostatic compression without any PTM were used on LTO. As shown in the XRD patterns in Fig. 2a (using helium as PTM) and Supplementary Fig. 3, available as Supplementary Data at *NSR* online (without any PTM), LTO underwent the same PIA transition under different hydrostaticity, confirming that the PIA is a intrinsic phenomenon for LTO. Figure 2b and c show the high-resolution transmission electron microscope (HRTEM) of the starting and recovered samples. The starting material is highly crystalline. However, the HRTEM image and its fast Fourier transform of the recovered sample all indicate a major amorphous structure. There is very little leftover nanopolycrystal (marked with the circle) dispersed in the major amorphous phase, in agreement with the XRD data. This further confirms the occurrence of the PIA in the sample and excludes the effects of pressure inhomogeneity on the observed phenomenon.

**Figure 3.**
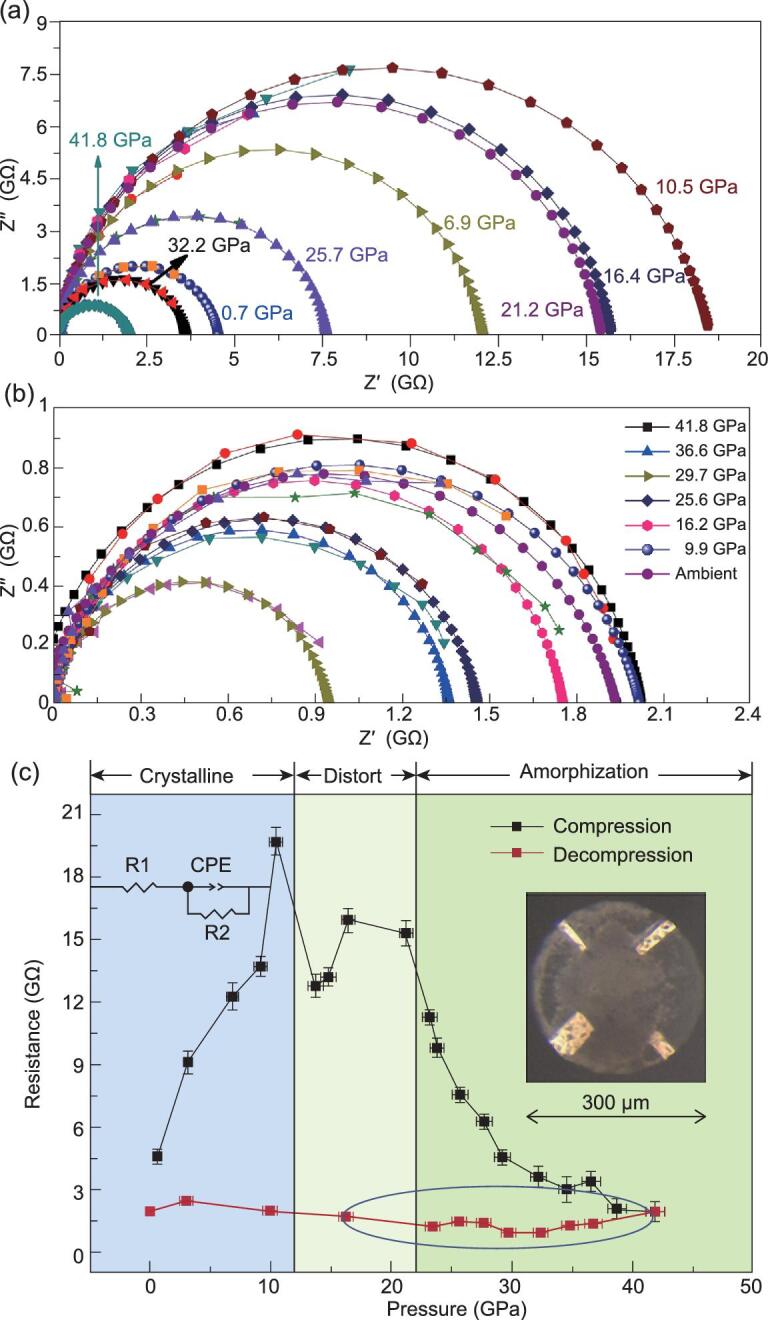
The Nyquist plots for various pressure values upon compression (a) and decompression (b). (c) The resistance changes with increasing and decreasing pressure; the inset figures show the corresponding equivalent circuit model and the four micro-electrode on the diamond culet.

High-pressure impedance spectroscopy was performed to investigate the transport properties of LTO during PIA. The Nyquist plots and fitted results using an equivalent circuit model are presented in Fig. 3a and b for the compression and decompression cycles, respectively. The plots show semicircles of different radii in the high-frequency regions. The equivalent circuit model is depicted in the inset of Fig. 3c, where *R*_1_ is the electrode contact resistance and CPE denotes the constant phase element that works as an alterable capacitance. The charge-transfer resistance *R*_2_ was determined by the semicircle deducted from the *Z*′-axis intercept, which was related to the resistance of bulk LTO. As shown in Fig. 3c, under compression, the starting resistance at 0.7 GPa is approximately 4.6 GΩ, which indicated the insulating nature of LTO. With increasing pressure, the resistance increased to a maximum of 19.6 GΩ at 10.5 GPa and then decreased as the lattice started to distort above 10 GPa. The resistance exhibited a clear decrease when amorphization began at approximately 25 GPa. It finally decreased to 1.9 GΩ at 41.8 GPa, which was much lower than the resistance in the initial state. We inferred that the high-pressure-induced amorphous LTO phase showed higher conductivity than the crystalline LTO. Upon decompression, the resistance of LTO at every pressure point maintained much lower resistance values in the range 0.94–1.4 GΩ, compared to the starting resistance of the crystalline LTO. Compared to crystalline LTO, we expect that the amorphous state of LTO with higher conductivity will perform better as a potential electrode material during the ionic and electron-transfer processes.

**Figure 4.**
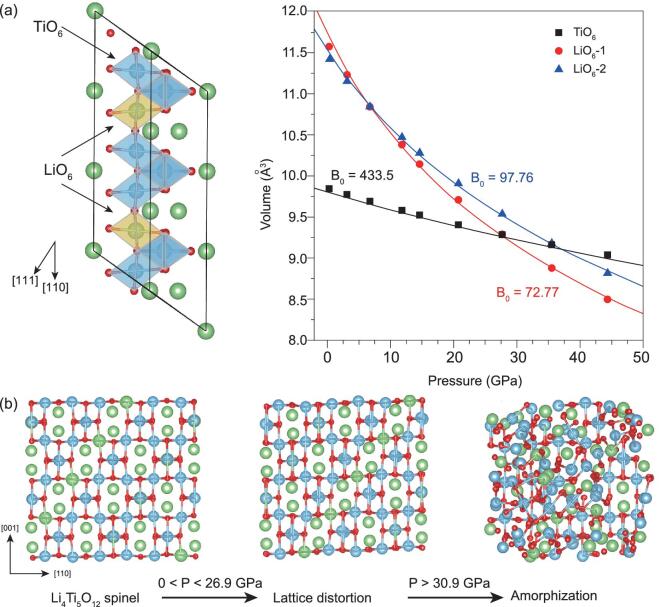
(a) The calculated pressure–volume relations and moduli of the TiO_6_ octahedron and LiO_6_ octahedron in position 1 and position 2 in the supercell. (b) Calculated structural transitions from LTO spinel at ambient pressure to lattice distorted LTO at a pressure above 26.9 GPa and subsequent amorphization above 30.9 GPa.

We fitted the pressure–volume relation with the third-order Birch-Murnaghan equation of state (EOS), as shown in the data plotted in Supplementary Fig. 5 of the Supplementary Material, available as Supplementary Data at *NSR* online. This bulk modulus obtained using the hydrostatic pressure condition is close to other lithium titanium oxides, such as LiTi_2_O_4_ and Li_2_Ti_2_O_4_ [25], indicating that the LTO spinel has similar compressibility. Yi *et al.* studied the structural properties of lithium titanium oxide using a density-functional-theory (DFT) plane-wave pseudopotential method [25], and suggested that the strength of  Ti–O bonds could determine the resistance of materials to uniaxial tensions in three directions. Therefore, the investigation of the bond-length variations between the Ti and O atoms is also important to understand the LTO bulk modulus. First-principles calculations based on DFT within the local-density approximation and the generalized gradient approximation were then carried out using the Vienna Ab Initio Simulation Package (VASP) [26–30]. Detailed calculation methods are provided in the Supplementary Material, available as Supplementary Data at *NSR* online. To understand the structural evolution and phase-transition mechanism of LTO under high pressure, we compared the volume changes of the TiO_6_ octahedra and LiO_6_ octahedra at positions 1 and 2 in the simulated model (1 × 3 × 1 supercell) presented in Fig. 4a. The results show that the octahedra volume changes in the [Li_1/3_Ti_5/3_]_16d_O_4_ framework display very different responses to the applied pressure. We fitted the pressure–volume data by an EOS to derive the corresponding moduli. The modulus of the LiO_6_ octahedra at position 1 is 72.7 GPa, which is 25.0 GPa lower than the modulus of LiO_6_ at position 2. Moreover, the calculated modulus of the TiO_6_ octahedron is 433.5 GPa, which is much higher than LiO_6_. The large discrepancies in the moduli for the different octahedra will directly result in different responses to high pressure, which eventually cause the structural distortion observed from the calculated high-pressure structure (Fig. 4b). The distorted lattice can cause the splitting and broadening of the diffraction peaks, as observed in the *in situ* XRD patterns. The distorted LTO was a metastable phase and could be fully restored when the pressure was released, while, at pressures above 30.0 GPa, the large distortion of the lattice might induce the collapse of the [Li_1/3_Ti_5/3_]_16d_O_4_ framework and lead to the final amorphization process.

**Figure 5.**
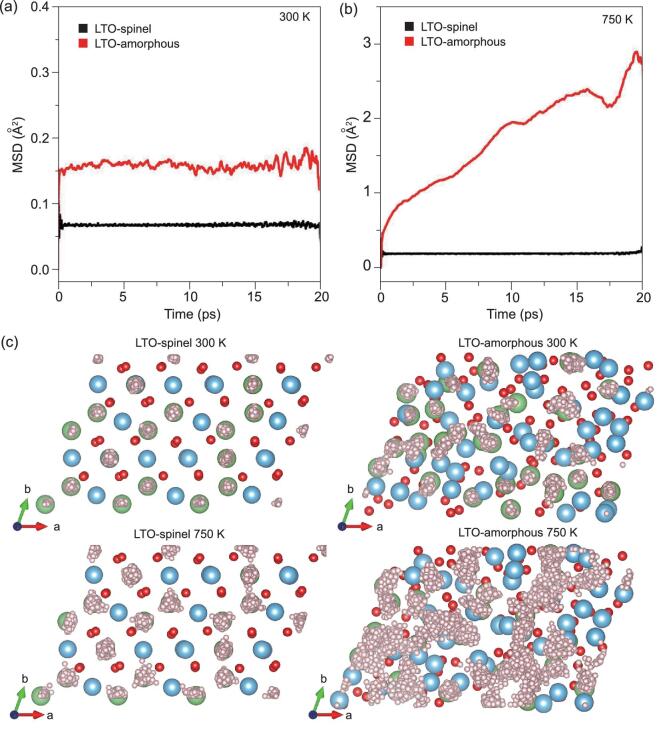
Mean square displacements of Li^+^ in crystalline and amorphous LTO at (a) 300 and (b) 750 K. (c) Trajectories of Li^+^ (small pink bullets) in LTO spinel and LTO-amorphous at 300 and 750 K.

The ionic transport properties of crystalline and amorphous LTO were investigated by first-principles molecular dynamics (FPMD) simulations to understand the significant enhancement of conductivity in the amorphous phase. The method is described in detail in the Supplementary Material, available as Supplementary Data at *NSR* online. As shown in Fig. 5a and b, the displacements of Li^+^ in LTO spinel are almost zero and show no increase with time, indicating no Li^+^ diffusion occurs in crystalline LTO at 300 and 750 K. On the other hand, the diffusion of Li^+^ in amorphous LTO is obvious, as shown by their trajectories (Fig. 5c). This simulation result is consistent with our impedance measurements, and this kind of increasing ionic conductivity by amorphization has also been reported in other amorphous materials used to fabricate batteries [31–33]. The amorphous phase induced by high pressure can promote Li^+^ diffusion and increase its ionic conductivity by providing defects for ion migration.

## CONCLUSIONS

In conclusion, a two-step structural evolution process of LTO upon compression was observed by *in situ* synchrotron XRD and *in situ* Raman spectroscopy. At high pressures of about 26.9 GPa, the inconsistent volume contractions of the LiO_6_ and TiO_6_ octahedra in the [Li_1/3_Ti_5/3_]_16d_O_4_ framework led to the structural distortion of the LTO spinel. The distortion was unstable and could be fully transformed back to the LTO-spinel structure when the pressure was released to ambient. When the pressure was further increased to 30.0 GPa, the distortion of the lattice caused the structural collapse of the [Li_1/3_Ti_5/3_]_16d_O_4_ framework, resulting in the initial phase transforming completely to an amorphous state. These results indicate that the different occupations of Li and Ti at the 16d sites in the LTO structure were responsible for the structural instability under high pressure. Simultaneously, amorphous LTO displayed better conductivity than crystalline LTO. These findings may offer a new idea for improving the conductivity of a LTO anode in LIBs using a high-pressure technique. Theoretical calculations revealed that the amorphous phase induced by high pressure can promote Li^+^ diffusion and increase its ionic conductivity by providing defects for ion migration. All of these findings increase the understanding of the structural and conducting properties of LTO and offer a new amorphous LTO phase that has the potential to become a novel anode material for LIBs.

## METHODS

LTO powder (99.9%, Alfa Aesar, USA), with particle sizes between 0.5 and 1 μm, was set in a Mao–Bell-type diamond anvil cell (DAC) with silicone oil as a PTM. *In situ* high-pressure XRD experiments using silicone oil and helium as a PTM were performed at beamline 15U1 of the Shanghai Synchrotron Radiation Facility (SSRF). The XRD measurements without PTM were carried out at beamline 10XU of SPring-8. The incident wavelengths of the beams at the two synchrotron X-ray sources were 0.6199 and 0.4136 Å, respectively. The microstructures of the recovered samples were investigated using a HRTEM, JEOL 2100F at the Center for Nanoscale Materials, Argonne National Laboratory. Pressure-dependent Raman spectra were detected using a Raman spectrometer (Renishaw inVia™, Renishaw plc, UK) with an excitation wavelength of 532 nm. Four-probe ac impedance spectroscopy measurements were performed by arranging four Pt electrodes on the diamond culet in the DAC loaded with the LTO sample. Micron-sized ruby chips were used as pressure markers (with an *R*_1_ fluorescence peak shift) throughout the experiments.

## Supplementary Material

Supplemental Files
