# Supplementary material for: Li-ion battery material under high pressure: amorphization and enhanced conductivity of Li4Ti5O12
Source: Natl Sci Rev. 2018 Oct 29;6(2):239–46. doi: 10.1093/nsr/nwy122 (PMC8291545; doi:10.1093/nsr/nwy122)
Supplement: Supplemental Files [file nwy122_supplemental_file.docx]

**Supplementary Materials**

Pressure makes better Li-ion battery material: amorphization and enhanced conductivity of Li_4_Ti_5_O_12_

Yanwei Huang^§,1,4^, Yu He^§,1,2,^, Howard Sheng^1,3^, Xia Lu^5^, Haini Dong^1,2^, Sudeshna Samanta^1^, Hongliang Dong^1^, Xifeng Li^6^, Duck Young Kim^1^, Ho-kwang Mao^1,7^, Yuzi Liu^8^, Heping Li^2^, Hong Li^9^, Lin Wang^*,1^

*^1^Center for High Pressure Science and Technology Advanced Research, Shanghai, 201203, China.*

*^2^Key Laboratory of High-temperature and High-pressure Study of the Earth’s Interior, Institute of Geochemistry, Chinese Academy of Sciences, Guiyang, Guizhou 550081, China.*

*^3^Department of Physics and Astronomy, George Mason University, Fairfax, Virginia 22030, USA.*

*^4^College of Materials and Environmental Engineering, Hangzhou Dianzi University, Hangzhou, 310018, China.*

*^5^State Key Laboratory of Organic-Inorganic Composites, Beijing Advanced Innovation Center for Soft Matter Science and Engineering, College of Energy, Beijing University of Chemical Engineering, Beijing 100029, China.*

*^6^School of Mechatronic Engineering and Automation, Shanghai University, Shanghai, 200072, China.*

*^7^Geophysical Laboratory, Carnegie Institution of Washington, , Washington, DC 20015.*

*^8^Center for Nanoscale Materials, Argonne National Laboratory, 9700 South Cass Avenue, Argonne, Illinois, 60439, United States*

*^9^Beijing National Laboratory for Condensed Matter Physics, Institute of Physics, Chinese Academy of Sciences, Beijing 100190, China.*

**Experiments**

Powder of LTO (99.9%) was purchased from Alfa Aesar without further purification. A Mao-Bell type diamond anvil cell (DAC) with 400 μm diameter culet was used to generate high pressure. LTO sample was loaded into a 135 μm diameter hole drilled in T301 stainless steel gasket which was pre-indented to a thickness of 45 μm. To produce a quasi-hydrostatic environment around the sample, we used silicone oil as a pressure transmitting medium. Pressure was determined by pressure dependent spectral shift of the sharp ruby fluorescence R1 line [R1,R2].The *in-situ* high pressure XRD experiments were performed at the 15U1 beamline of Shanghai Synchrotron Radiation Facility (SSRF). The incident wavelength of the beam was 0.6199 Å with a beam size of 2×7 µm^2^. The high purity CeO_2_ powder was used to calibrate the geometric parameters. The FIT2D software was used to integrate the diffraction rings into one-dimensional patterns. The XRD patterns were analyzed with Rietveld refinement using the GSAS program package with a user interface EXPGUI [R3,R4]. *In-situ* high pressure Raman scattering measurements were performed using a Renishaw inVia Raman spectrometer with excitation wavelength of 532 nm.

**Rietveld refinements**

FIG. S1(a) shows the XRD pattern of LTO at ambient conditions with Rietveld refinement results based on a previously reported mode [R5].The short tick lines below the diffraction pattern indicate the locations of the calculated diffraction peaks in the plot. The residual difference between the calculated and the experimental patterns is shown at the bottom of the plot. A good refinement result confirms that the crystal structure of LTO at ambient conditions is Fd$\bar{3}$m(S.G.: 227), as shown in FIG. S1(b). The lattice constant is calculated to be 0.835 nm with 3.5% of Rp value after refinement.


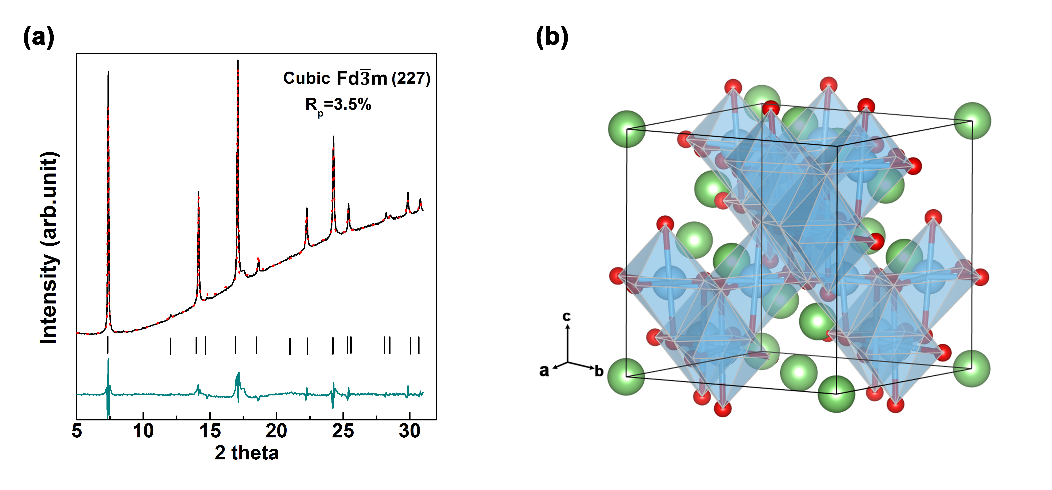


FIG. S1. (a) Refinement pattern of the LTO XRD at ambient conditions. Measured (black lines) and calculated (red dashed lines) patterns are shown, together with the difference curve (gray green lines) and calculated positions of Bragg reflections (tick marks). (b) LTO-spinel structure. The green spheres indicate Li atoms, and red for O atoms, blue for Ti atoms. The blue octahedrons construct the [Li_1/3_Ti_5/3_]_16d_O_4_ framework.

**DFT calculations**

In order to clarify the splitting of the diffraction peaks, we performed further DFT calculations on LTO with a supercell containing 166 atoms. It is noted that the structural relaxation was conducted at different cell volume without any confinement on the symmetry. By fitting with a Birch−Murnaghan equation of state (EOS), we obtained the optimized structure of LTO at different pressures. The obtained structure start to distort at ~8.2 GPa, as shown in FIG. S2(a). We transferred these structures into powder XRD patterns (FIG. S2 (b)). The (111) peak splitting can be clearly observed in these patterns at high pressure, which is consistent with our experimental results. Moreover, other peaks also spited to several peaks at high pressure, which corresponded to the broadening of XRD peaks in our experimental results. The different peak splitting pressure between experiment and calculation was caused by the limited of the supercell and the ordering of Li/Ti occupancy at 16d sites. As we got the distorted structure of LTO by DFT calculation, it is easier to understand the peak splitting and lattice distortion nature of LTO under high-pressure. As shown in FIG. S2 (a), it shows the layered structure of LTO. In the structure, there are four different layers: layer a is composed by LiO_6_ octahedral at both 8a and 16d sites; layer b is composed only by TiO_6_ octahedral at 16d site; layer c is composed LiO_6_ octahedral at 8a site and TiO_6_ octahedral at 16d site; and layer d is composed by LiO_6_ and TiO_6_ octahedral at 16d site. As we described in the manuscript, the compressibility of LiO_6_ and TiO_6_ at 16d site is quite different. LiO_6_ octahedral is much softer than TiO_6_ octahedral. In this way, layer a should be softer than layer c, and layer d should be softer than layer b. As a result, the distance of each (111) layers changed with pressure and leaded to the (111) peak splitting under high pressure. The case should be the same for the distances of other planes, which eventually leads to the broadening of diffractions peaks.


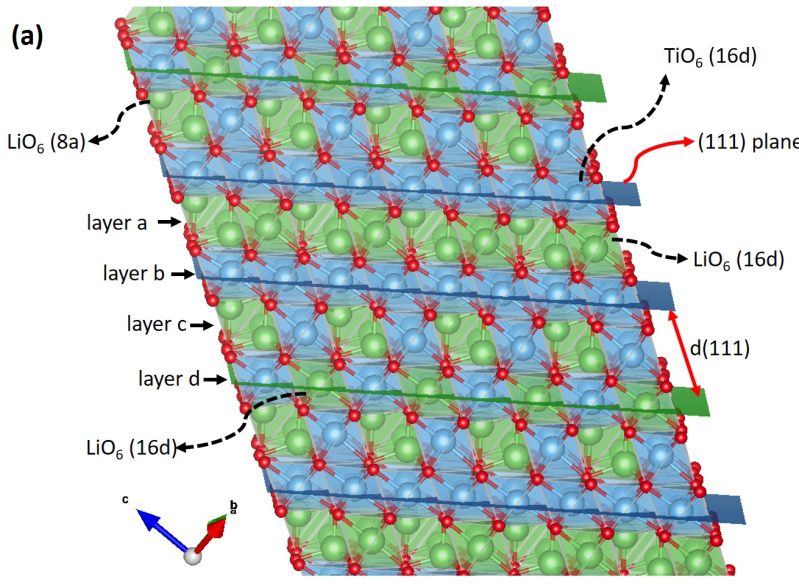

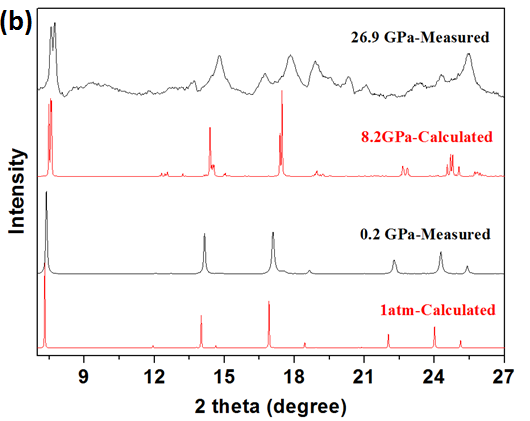


FIG. S2. A supercell containing 166 atoms of our DFT calculation shows slight distortion at 8.2 GPa (a). Comparison of XRD patterns of experiments and calculations.

**Pressure dependence of the Raman shift**

As shown in FIG. S3, all the five Raman peaks, assigned to two F_2g_ modes, one E_g_ mode and two A_1g_ mode, shift to higher frequencies under compression. The pressure dependence of the Raman frequencies shift slope had been fitted linearly over the compression. The fitted values corresponded to the peaks of F_2g_ (234 cm^-1^), F_2g_ (344 cm^-1^), E_g_ (425 cm^-1^), A_1g_(674 cm^-1^) and A_1g_ (759 cm^-1^) modes are 2.51 cm^-1^/GPa (0.09), 1.3 cm^-1^/GPa (0.07), 2.79 cm^-1^/GPa (0.09), 2.94 cm^-1^/GPa (0.07), and 2.16 cm^-1^/GPa (0.13), respectively.

The intensities of the peaks decrease gradually at low pressure range, and decrease sharply as the pressure above ~21 GPa, suggesting the pressure-induced distortion starts.


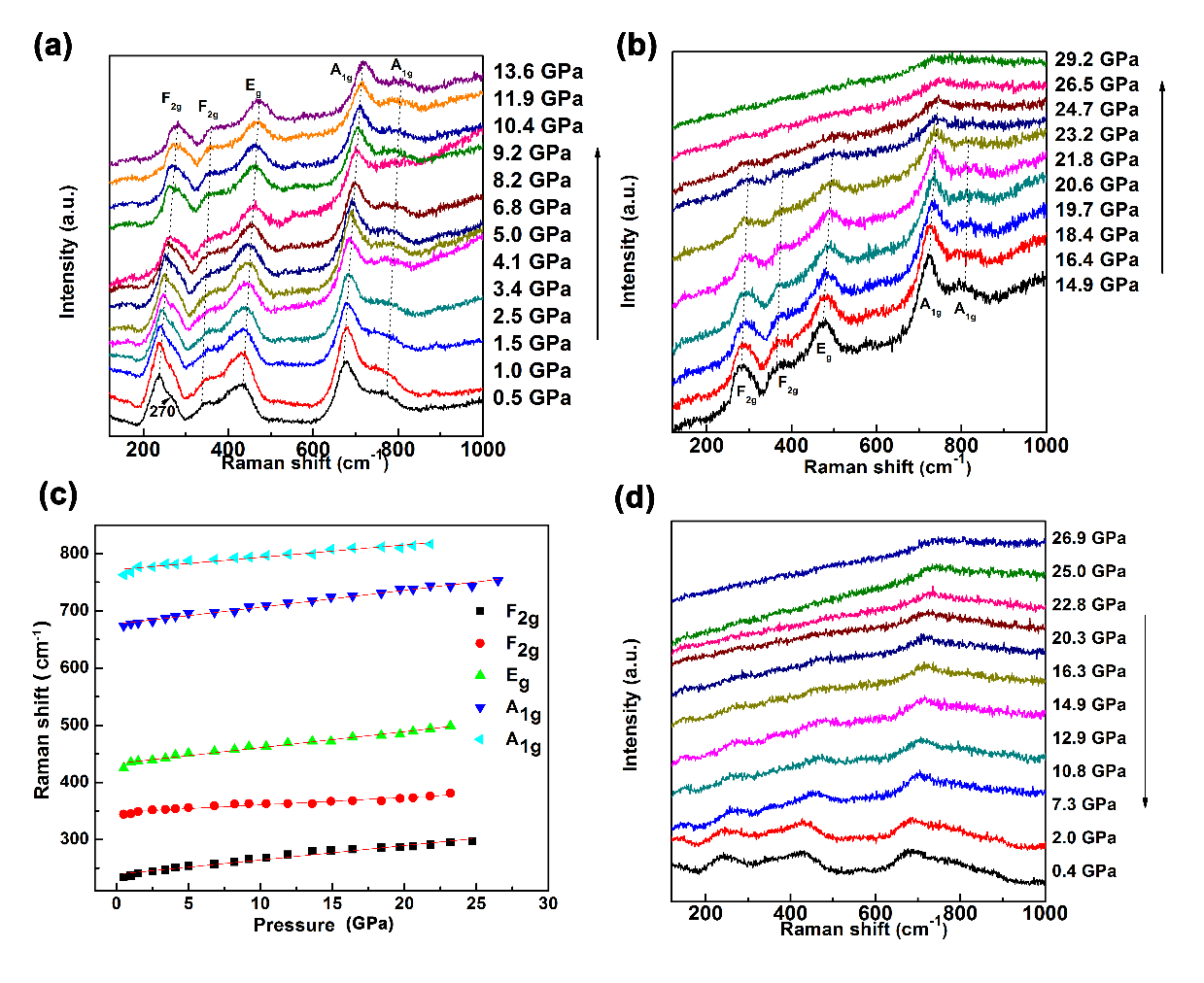


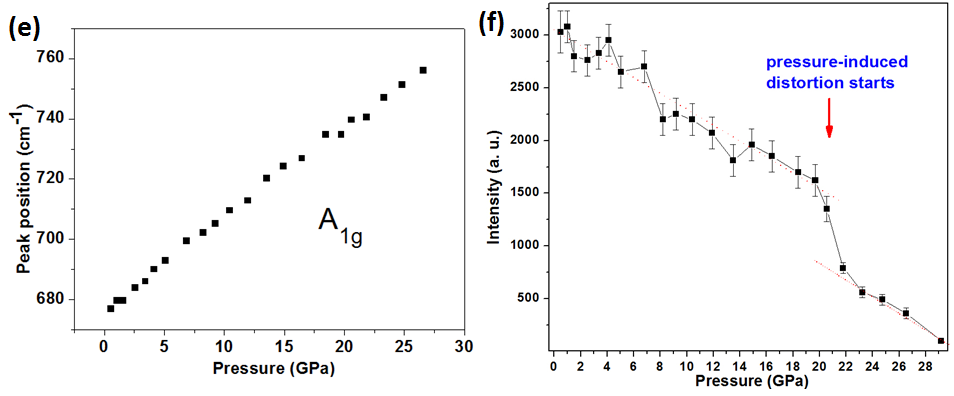


FIG. S3. *In-situ* Raman spectra of the LTO-spinel upon compression at the range of (a) 0.5 ~ 13.6 GPa and (b) 14.9 ~ 29.2 GPa. (c) Pressure dependence of vibrational frequencies observed of LTO-spinel in the low frequency range of 100 ~ 1000 cm^-1^ at room temperature.(d) *In-situ* Raman spectra of the LTO-spinel upon decompression at the range of 26.9 GPa ~ 0.4 GPa. (e) Zoom-in of pressure dependence of A1g peak, showing linear shift. (f) Peak intensity of A1g peak shows a sharp decrease at ~21 GPa.

**Compression with different PTM**

Fig. S4 and S5 show the XRD and Raman spectra of LTO compressed without any pressure medium and using helium as medium, respectively. All the data confirmed that LTO underwent PIA under both non-hydrostatic and hydrostatic compression conditions, confirming the PIA is general phenomenon in LTO.


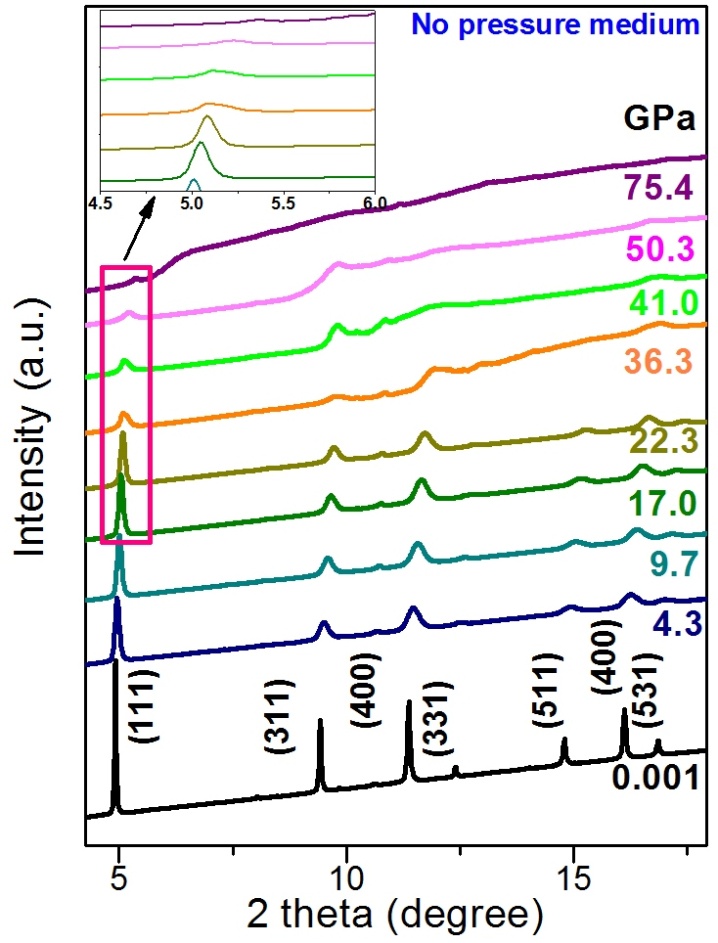


FIG. S4. *In-situ* synchrotron XRD patterns and of LTO-spinel under nonhydrostatic compression without any PTM.


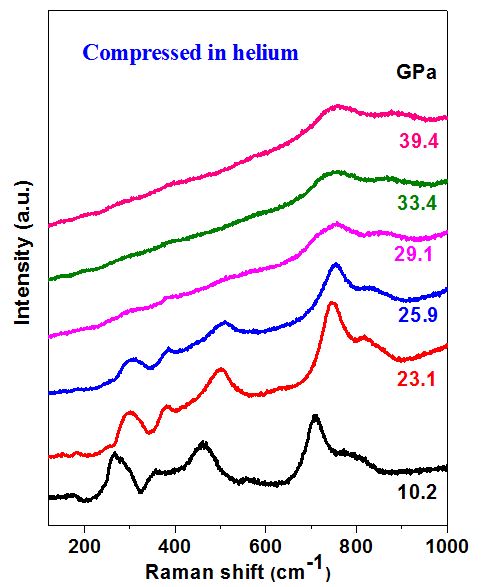


FIG. S5. *In-situ* Raman spectra of LTO-spinel under hydrostatic compression using Helium as PTM.

**First-principles calculations**

First-principles calculations were performed based on the density functional theory (DFT)[R6,R7]within the local density approximation (LDA) and generalized gradient approximation (GGA) [R8,R9].All total energy calculations were carried out using the Vienna Ab Initio Simulation Package (VASP) [R10].In our calculations, a plane wave representation for the wave function with a cut off energy of 540 eV were adopted. Geometry optimizations were performed by a conjugate gradient minimization until all the forces acting on ions were less than 0.01 eV/Å per atom. The calculation model was a 1×3×1 supercell based on the primitive cell of LTO. The calculated LTO model had a minimum energy after carefully optimizing the arrangements of Li ions in 16*d* sites[R11,R12].The fully relaxed structure was treated as the structure at zero pressure. K-point mesh with a spacing of *ca.* 0.03 Å^−1^ was adopted. The relaxed structure calculations were performed at various constant volumes and the energy−volume data were fitted to a third-order Birch−Murnaghan equation of state (EOS):

$E\left( V \right)=E_{0}+\frac{9V_{0}B_{0}}{16}\left\{ \left[ \left( \frac{V_{0}}{V} \right)^{\frac{2}{3}}-1 \right]^{3}B_{0}^{'}+\left[ \left( \frac{V_{0}}{V} \right)^{\frac{2}{3}}-1 \right]^{2}\left[ {6-4\left( \frac{V_{0}}{V} \right)}^{\frac{2}{3}} \right] \right\} (1)$,

where E_0_ is denoted as the intrinsic total energy at zero pressure, V_0_ is the equilibrated volume at zero pressure, B_0_ is the bulk modulus and B_0_’ the first pressure derivative of the bulk modulus. The relation between the pressure and the volume at zero Kelvin degree can be expressed as:

$$P\left( V \right)=\frac{3B_{0}}{2}\left[ \left( \frac{V_{0}}{V} \right)^{\frac{7}{3}}-\left( \frac{V_{0}}{V} \right)^{\frac{5}{3}} \right]\left\{ 1+\frac{3}{4}(B_{0}^{'}-4)\left[ \left( \frac{V_{0}}{V} \right)^{\frac{2}{3}}-1 \right] \right\}\left( 2 \right).$$

The cell volumes of LTO at different pressures are plotted as black dots in FIG. S6. They were fitted to the third-order Birch-Murnaghan equation of state as shown in this method. The experimental value was close to the prediction made using the GGA. The little discrepancy in the GGA prediction was probably due to the method itself and its overestimation of cell volumes [R13]. Overall, the calculated bulk moduli were slightly smaller since the LTO structure was mainly constructed by the [Li_1/3_Ti_5/3_]_16d_O_4_ framework, and this discrepancy was likely caused by different Li and Ti occupations at the 16*d* sites.


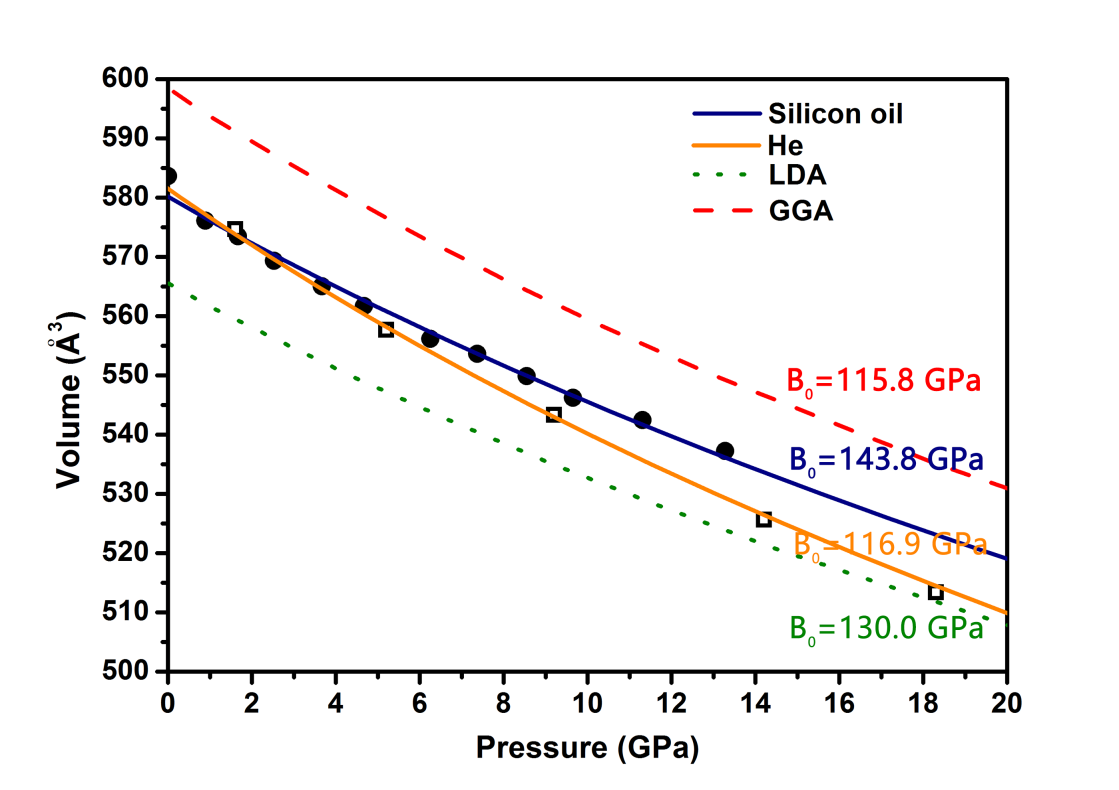


FIG. S6. The calculated and experimental pressure-volume diagram for the LTO-spinel. The black dots were obtained by *in-situ* synchrotron XRD. The fitted bulk moduli are denoted as well.

In order to further clarify the mechanism of structural evolution and phase transition of LTO under high-pressure, the calculated Li-O and Ti-O bond length variations and the corresponding volumes of theLiO_4_ tetrahedron, TiO_6_ octahedron and LiO_6_ octahedron under pressure are demonstrated in FIG. S7. The bond length and volume decrease with the applied pressure, and Li_16d_-O bondsare much easier to be compressed with respect to Ti_16d_-O bonds. The volume of the LiO_6_ octahedron shows over 18% more reducing compared with the TiO_6_ octahedron volume at 30 GPa. It suggeststhat the octahedralvolume changesin [Li_1/3_Ti_5/3_]_16d_O_4_ framework display much different responses to applied pressure.


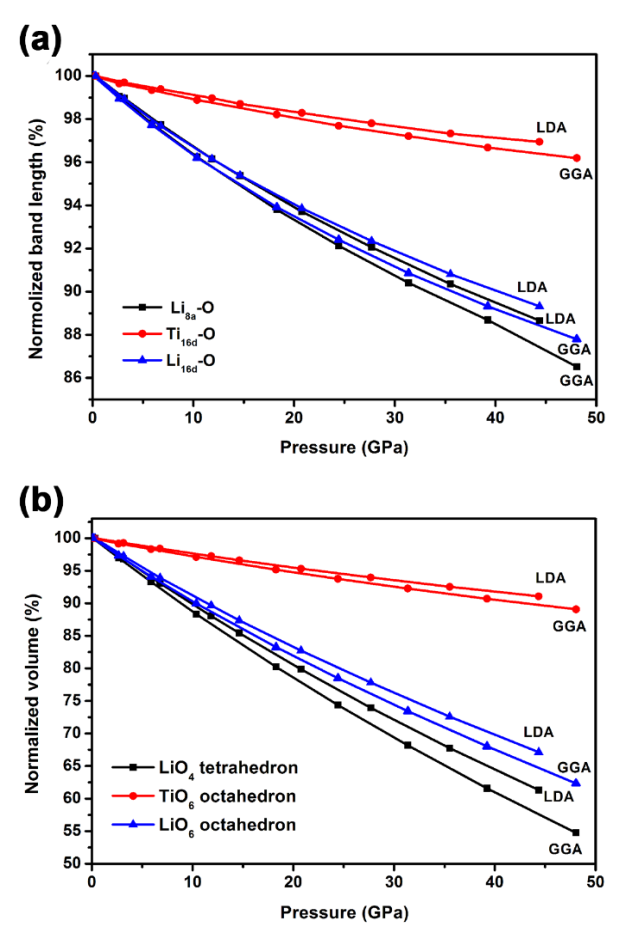


FIG.S7. (a)The normalized bond length variations of the Li_8a_-O, Ti_16d_-O and Li_16d_-O bonds and (b) normalized volume changes of the LiO_4_ tetrahedron, TiO_6_ octahedron and LiO_6_ octahedron under high-pressure.

**First-Principles Molecular Dynamics (FPMD) calculations**

In First-Principles Molecular Dynamics (FPMD) calculations, a 3× 2× 2 (168 atoms) supercell of primitive LTO cell was employed. The energy cutoff is reduced to 400 eV. To keep the computational cost manageable, the Brillouin zone sampling was performed at the Γ point. Amorphous LTO structure was prepared by FPMD method. The crystalline LTO supercell was heated above its melting temperature (3000 K) for 8 ps. Then the system is gradually quenched at 2500, 2000, 1500, 1000, 750, 500, and 300 K for 4 ps to obtain the final amorphous phase at room temperature [R14, R15]. To calculate mean square displacement, all the models were thermally equilibrated for 2 ps then followed by a MD run at different temperatures (300 to 1000 K) for 20 ps. Time average mean square displacement (MSD)[R16] of the different atoms are generated using the atomic configuration information from every finite MD time step defined as:

$$\left\langle\left[ \vec{r}\left( t \right) \right]^{2} \right\rangle=\frac{1}{N}\sum_{i=1}^{N} \left\langle\left[ \vec{r_{i}}\left( t+t_{0} \right)-\vec{r_{i}}\left( t_{0} \right) \right]^{2} \right\rangle$$

$\vec{r_{i}}\left( t \right)$ is the displacement of the Li ion at time t, and N is the total number of Li ions in the system.

**Reference**

[R1] Dylla, A. G.; Henkelman, G.; Stevenson. Accounts Chem. Res. **2013** 46 1104-1112.

[R2] Zhang, J.; Xi, J.; Ji, Z. J. Mater. Chem. **2012**, 22, 17700.

[R3] Larson, A. C.; von Dreele, R. B. Los Alamos National Laboratory Report LAUR **2004**, 86-748.

[R4] Toby, B. H. EXPGUI. J. Appl. Crystallogr. **2001**, 34, 210–213.

[R5]Deschanvres, A.; Raveau, B.; Sekkal, Z.  Mater. Res. Bull. **1971**, 6, 699-704.

[R6] Hohenberg, P.; Kohn, W. Phys. Rev. B **1965**, 136, 864–871.

[R7] Kohn, W.; Sham, L. J. Phys. Rev. A **1965**, 140, 1133–1138.

[R8] Ceperley, D.; Alder, B. Phys. Rev. Lett. **1980**, 45, 566–569.

[R9]Perdew, J. P.; Zunger, A. Phys. Rev. B **1981**, 23, 5048–5079.

[R10] Kresse, G.; Furthmüller, J. Phys. Rev. B **1996**, 54, 11169-11186.

[R11] Sun, Y.; Zhao, L.; Pan, H. L.; Lu, X.; Gu, L.; Hu, Y.-S.; Li, H.; Armand, M.; Ikuhara, Y.; Chen, L. Q.; Huang, X. Nat. Commun. **2013**, 4, 1870.

[R12] Mo, Y.; Ong, S. P.; and Ceder, G. **2012**, 24, 15–17.

[R13] D. Santamaria-Perez et al., Inorg. Chem. **2012**, 15, 5779-5786.

[R14] P. Johari, Y. Qi, V. B. Shenoy, Nano Lett. **2011**, 11, 5494–5500.

[R15] J. Pan, Q. Zhang, J. Li, M. J. Beck, X. Xiao, Y.-T. Cheng, Nano Energy, **2015**, 13, 192-199.

[R16] Y. Mo, S. P. Ong, G. Ceder, Chem. Mater. **2012**, 24, 15.
